# Factors Associated with Low Bone Density in Opioid Substitution Therapy Patients: A Systematic Review

**DOI:** 10.7150/ijms.52201

**Published:** 2021-01-01

**Authors:** Fitri Fareez Ramli, Syed Alhafiz Syed Hashim, Nadia Mohd Effendy

**Affiliations:** 1 Department of Pharmacology, Faculty of Medicine, Universiti Kebangsaan Malaysia, 56000 Cheras, Kuala Lumpur, Malaysia.; 2 Faculty of Medicine and Health Sciences, Universiti Sains Islam Malaysia, Persiaran Ilmu, Bandar Baru Nilai, 71800 Nilai, Negeri Sembilan, Malaysia.

**Keywords:** Opioid, Methadone, Buprenorphine, Bone mineral density, Bone mass

## Abstract

**Background:** Long-term opioid therapy is a risk factor for low bone mineral density (BMD). However, other factors may also contribute to low BMD. Several studies have examined the variables that might contribute to low BMD in patients receiving opioid replacement therapy (OST). However, to our knowledge, there was no systemic review conducted to address this particular issue. Thus, we reviewed the articles on the factors associated with low BMD in the population of opioid use disorder receiving substitution therapy.

**Methods:** The articles that examined correlates or risk factors of low BMD in OST population were retrieved from OVID, SCOPUS, and PUBMED from inception until July 2020 by two independent investigators.

**Results:** A total of 429 articles from three databases were retrieved initially. After screening based on eligibility criteria, five articles were included in the final analysis. The risk factors or correlates found to be significantly associated with low BMD in the OST population include male gender, low body mass index, low testosterone level, methadone or heroin use, and longer duration of heavy alcohol use. The review limitations include small sample sizes and inconsistent definition of variables.

**Conclusion:** OST patients should be screened for BMD and its associated factors. Guidelines and training of practitioners involving in the OST service should be provided to increase the detection of low BMD in the OST population.

## Introduction

Opioids are drugs that are widely used for pain management. It is an essential drug listed by the World Health Organization for the treatment of cancer and non-cancer-related pain as well as management of psychoactive substance use disorder 1. Given the intensely pleasurable effect of opioids secondary to reward system activation, it is often abuse for recreational use 2. However, prolonged use of opioids for chronic diseases or recreational use possesses a significant threat toward opioid dependency and addiction 2. The estimated global prevalence of opioid dependency in 2017 was 40.5 million, more than double increment than those reported in 2010 3, 4. Although its development is not definite, extra-medical use of opioids contributes towards a significantly higher risk for opioid dependency compared to medical use, with the former affecting up to two-thirds of patients than less than one-third in the latter population 4.

Opioid substitution therapy (OST) is the long-term, most effective treatment for opioid use disorder 4, 5. Methadone and buprenorphine are two drugs commonly used in OST. Methadone is an opioid agonist and has been proven to be more cost-effective than other treatment measures, such as buprenorphine 6, 7. On the other hand, buprenorphine is a partial opioid agonist that is available as an alternative to methadone maintenance treatment (MMT). Despite the positive impact of OST in patients, OST is also associated with unwanted treatment effects such as sexual dysfunction, disturbed menstruation, infertility, and low bone mineral density (BMD) 8-15. These adverse effects may develop as a consequence of opioid-induced hypogonadism or other-related mechanisms 15.

Pre-clinical studies had reported a direct inhibitory effect of the opioid on the hypothalamus, pituitary glands, and testes. The inhibitions result in the disturbance of gonadotropin-releasing hormone, luteinizing hormone, and follicle-stimulating hormone release, resulting in a secondary decreased in serum testosterone levels 16-18. Numerous clinical studies had reported a high prevalence of hypogonadism in the MMT population, ranging from 35.1-40.8% 19, 20. Furthermore, testosterone levels were found to be significantly lower in the MMT population than in the general population and patients on buprenorphine maintenance therapy (BMT) 11, 19, 21. On the other hand, some studies reported a negligible difference in plasma testosterone levels between BMT and the control group 19, 21. Low testosterone level is one of the primary predictors of BMD in general and OST populations 10, 22, 23. Similarly, the use of opioid as analgesics have been found to be associated with a remarkable reduction in BMD 23, 24, probably owing to hypogonadism 25, 26. Several studies had reported significant improvement of BMD in men with low testosterone levels receiving testosterone replacement therapy 27, 28. This finding indicates a significant contribution of testosterone in bone health maintenance.

Other than opioid-induced hypogonadism, the direct inhibitory action of the opioid on bone is another potential mechanism of bone pathology in opioid dependence population. This mechanism is supported by the presence of opioid receptors (mu, delta, and kappa) and opioid growth factor receptor on osteoblast 29, 30. Evidence of enhanced osteocalcin synthesis, osteoblast activation, and increase in bone mineralization following administration of naltrexone, an opioid antagonist, further supported the role of opioid in bone formation 29, 30.

The prevalence of low BMD in the OST population is generally higher than the general population, ranging from 34-83 % 10-13, 31. Various risk factors for low BMD in OST patients have been identified, which include male gender, lower weight, heavy alcohol use, and low testosterone levels 10, 12, 14. Given the high prevalence of low BMD in the OST population, particularly in the MMT population and less attention received by these specific populations, crucial clinical factors associated with low BMD must be defined to guide a clinician who mainly involves in OST service. This is essential to prioritize the screening for low BMD in the OST population. To our knowledge, there is no systematic review conducted before, which focuses on the OST population for the clinical factors associated with low BMD. The review aims to determine the risk factors for low BMD in the OST population.

## Materials and Methods

We included all observational studies such as case-control, cross-sectional, and cohort studies evaluating the risk factors or correlates for low BMD. We excluded abstracts, case reports, case series, and reviews.

### Target population

We included all patients on OST treatments, which included patients receiving MMT and BMT. All patients must be at least 18 years old. No restriction in terms of sex and ethnicity. MMT is defined as a patient receiving methadone therapy for an indication of opioid use disorder or opioid addiction. BMT is defined as a patient receiving buprenorphine therapy for the indication of opioid use disorder or opioid addiction. We excluded the use of opioids for both cancer and non-cancer pain.

### Outcome

BMD as measured by dual-energy X-ray absorptiometer or quantitative ultrasound at any sites including, total hip, femoral neck, greater trochanter, lumbar spine, whole body, and radius (mid or distal).

### Search methods for identification of studies

#### Database search

**Figure 1:**
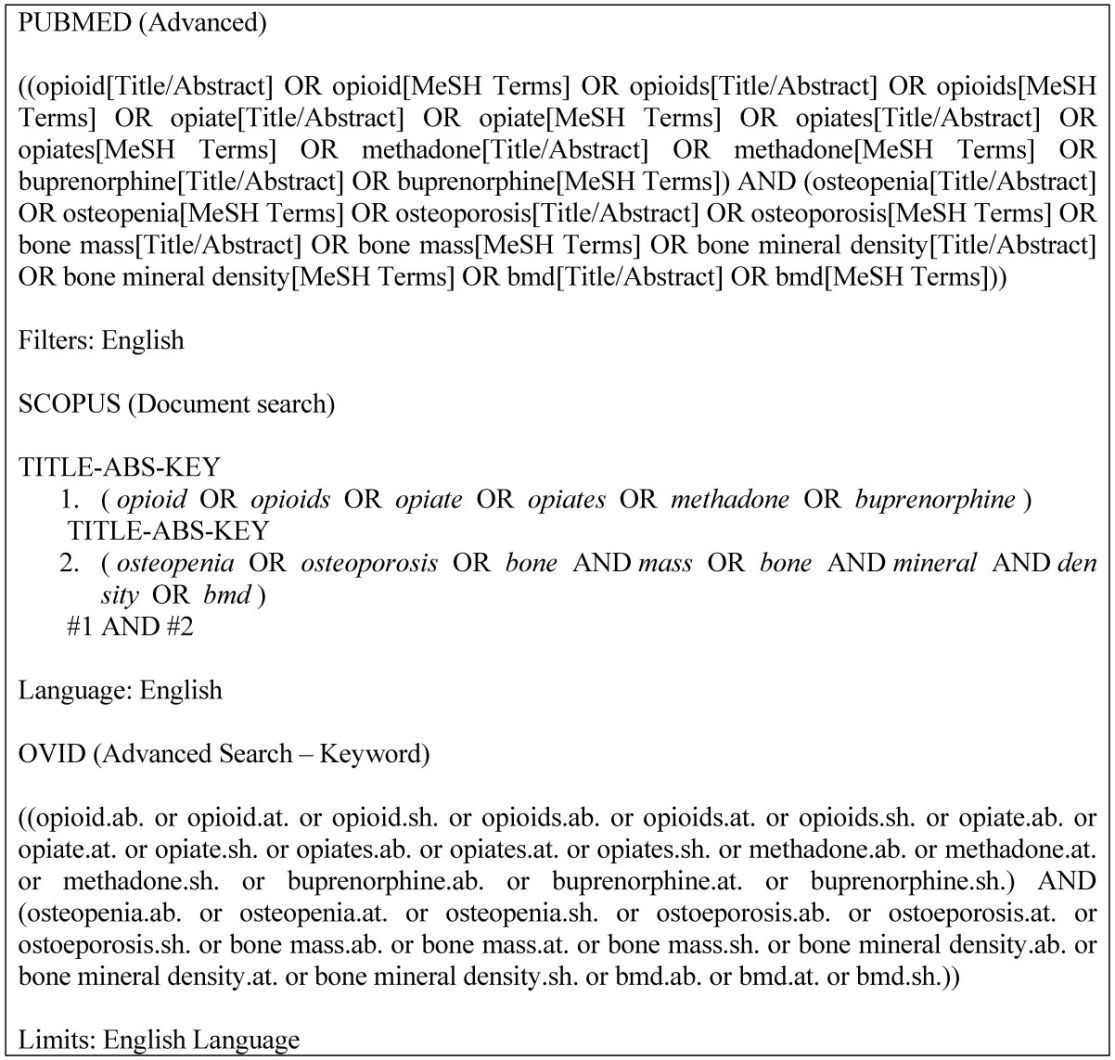
Search strategies for all databases.

We searched using PUBMED, OVID, and EMBASE databases from inception to July 2020 for observational studies. The studies included cohort, cross-sectional, and case-control studies describing the association between the risk factors or correlates for low BMD in OST patients. We had limited our searches to the English language. We used the following search terms: (“opioid” OR “opioids” OR “opiate” OR “opiates” OR “methadone” OR “buprenorphine”) AND (“osteoporosis” OR “osteopenia” OR “bone mass” OR “bone mineral density” OR “BMD”). The search strategy for each database can be referred to as Figure 1.

#### Selection of studies

The selections of the studies were conducted by FFR and NME independently based on three phases. Firstly, the articles were screened, and those that did not match the inclusion criteria solely based on their titles were excluded. Secondly, all the abstracts of the remaining articles were screened, and papers that did not match the inclusion criteria were excluded. Lastly, the remaining articles were read to exclude the second group of articles that did not match our inclusion criteria. The remaining papers were screened again by the FFR and NME before the data extraction phase. Any discrepancy between the FFR and NME were resolved by discussion or by involving a third author, SASH, as an adjudicator.

#### Data extraction and quality assessment

The extraction of the data was conducted by FFR and NME independently. The studies retrieve from the database are initially screened for eligibility based on the inclusion criteria mentioned above. We did not contact authors of published articles due to feasibility reason. FFR and NME independently extracted the following data: the first author's name, year of publication, types of study, study regions, sample size, patients' demographic characteristics (gender, age), and associated risk factors. We assessed the potential risk of bias in studies based on the modified Newcastle-Ottawa scale for cross-sectional 32 and cohort studies 33.

## Results

**Figure 2:**
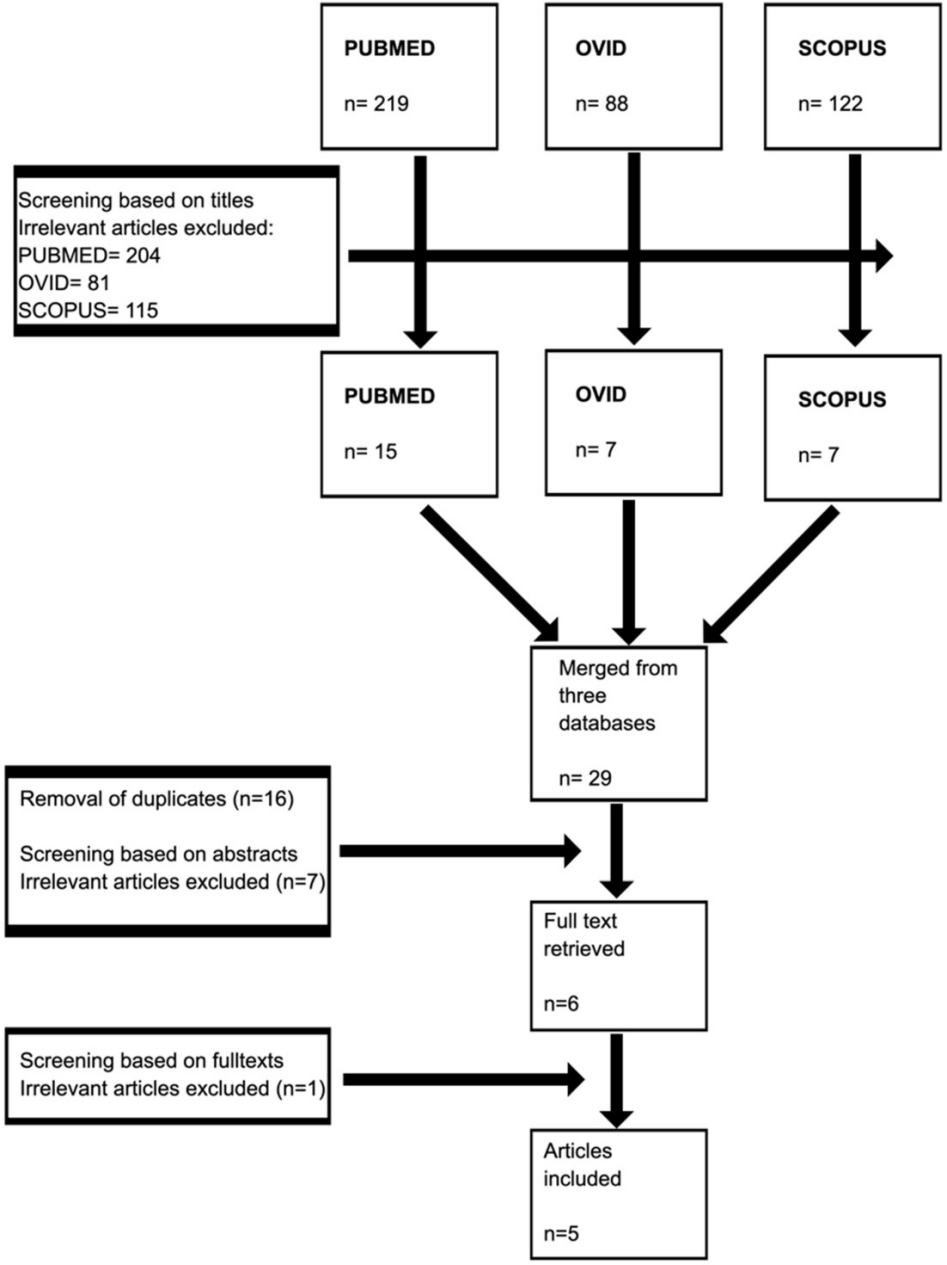
Selection process of the studies.

A total of 429 articles from three databases were retrieved in the first phase. Removal of irrelevant articles from each database yielded a total of 29 articles. These articles were grouped, and 16 duplicates were removed. The remaining articles were screened based on abstract, and seven were removed. Six articles were screened for full text, and only five articles were included in the final analysis (Figure 2). Two studies were conducted in the US, two studies in Switzerland, and one study in New Zealand. Four studies consisted of MMT population, and only one study had other forms of OST (Table 1).

### Risk of bias assessment

The score of the Newcastle-Ottawa scale for cross-sectional studies ranged between four to six, with a mean score of 4.75. The score of the Newcastle-Ottawa scale for cohort study was five.

### Risk Factors for Low Bone Mineral Density

The risk factors or correlates found to be significantly associated with low BMD in the OST population include male gender, low BMI (underweight), low testosterone level, methadone, or heroin use and longer duration of heavy alcohol use. Other factors, such as age and smoking, were not significantly associated with low BMD.

## Discussion

We found that four studies had cross-sectional or case-control designs and one cohort study. Three studies had less stringent criteria that excluded only those who were not willing to give or withdrew the consent 10, 11, pregnant, or weight over 300 lb (equivalent to 136.1 kg) 12. One study had stringent criteria that excluded HIV-infected and underweight patients, as these may become confounding factors for BMD 13.

In terms of opioid treatment, three studies had a homogenous population, which consisted of only the MMT population 11-13. Gotthardt et al. 10 conducted a study in the OST population that consisted of MMT, BMT, and morphine sulfate replacement therapy patients. In contrast, Sharma et al. 14 study population only consisted of one-third of the MMT population.

Two studies had reported a significantly lower BMD in the male MMT population compared to age-matched and weight-matched male control 11, 12. Gotthardt et al. 10 also reported a significant difference in terms of BMD between OST patients and control. However, the analysis conducted did not stratify into three groups (BMT, MMT, and morphine), so specific treatment effects on BMD could not be concluded as different OST may have different effects on BMD. Analyses of opioid intake factors such as duration and dose of either total opioid or OST intake were not significantly associated with BMD 10, 34. In the female population, the difference between the MMT population and control was not significant 11, 13. In contrast, a prospective cohort study of women with or at risk of HIV reported methadone use was one of the factors significantly associated with lower BMD 14. The differing result may be explained in terms of methodologies. Both Grey et al. 11 and Milos et al. 13 were case-control studies, comparing the BMD between OST and healthy controls. Sharma et al. 14, on the other hand, conducted a prospective cohort study of women in the menopause study cohort who were at-risk or infected with HIV.

Two studies reported that the BMD of the male MMT population was significantly lower when compared to the female MMT population 11, 34. Other studies had a homogenous population (either all men or women), so the effects of gender could not be determined 10, 13, 14. One possible explanation is opioid-induced hypogonadism, as testosterone is the primary determinant of bone formation 35. Out of six included studies, only two studies had measured the levels of testosterone. However, only one study had analysed the relationship between testosterone levels and BMD. Gotthardt et al. 10 in a study in Switzerland reported that free testosterone levels had a positive association with lumbar BMD in a multivariate analysis. Stratification of free testosterone levels based on quartiles showed that patients with free testosterone levels of ≥135 pmol/L had significantly higher BMD than those with levels of <135 pmol/L. On the other hand, Grey et al. 11 did not analyze the association between testosterone levels and BMD but reported significantly lower total testosterone levels in the male MMT population compared to age-matched healthy controls. The low testosterone levels might be attributed to opioid suppressive effects at the hypothalamus, pituitary gland or gonadal levels, resulting in hypogonadism 16-18, 36. Other possible explanation is the effects of opioid on bone cells, such as osteoblast and osteocytes.

In terms of BMI, one study reported a significant association between BMI and BMD in men on OST 10. The results remained significant in multivariate analysis. The underweight group had significantly lower BMD, while the overweight group had significantly higher BMD compared to the normal BMI group. Another study also reported a similar finding 14. The results were similar to other studies in the general population 37-41. The mechanism of increased BMD in overweight or obese patients might be attributed to the increase in mechanical loading as well as elevated estrogen, leptin, and insulin, which induce osteogenesis and inhibit bone resorption 42.

One study had reported that a longer duration of heavy alcohol consumption was associated with lower BMD 34. Similarly, Kim et al. 43 reported a significant association between chronic alcohol intake among patients without liver disease. In contrast, Grey et al. 11 reported that current alcohol use was not associated with BMD in multiple regression analyses. The different findings might be attributed to the differences in terms of the definition of alcohol use and analyses conducted. Kim et al. 34 investigated the association between the duration of heavy alcohol use and BMD. They defined heavy alcohol use as persistent alcohol use for more than three drinks on more than three occasions per week for at least one year. On the other hand, Grey et al. 11 defined recent alcohol use loosely, without any pre-specified amount, frequency, and duration. The effects of alcohol on BMD may be different, depending on the amount of alcohol intake. Multiple studies in other population had reported an improvement in BMD with moderate alcohol intake 44-47.

The generalisability of the included studies were limited due to several factors: 1) small sample size; 2) recruitment from a single center; 3) some had stringent eligibility criteria; 4) different exposure (correlates) definition and selection. These factors hindered meta-analysis for the current study. The majority of the studies were cross-sectional, hindering the temporal association from being made. Moreover, the primary OST type in the included studies was MMT, and no specific analysis was conducted to clarify the effect of other forms of OST treatment such as BMT. Despite numerous limitations, this review gives insights on the potential risk factors and may enhance further research on this under-researched field. The direction of the future studies should emphasize on larger sample size, the replication of the study in different populations (other regions), cohort studies, the study of effects of other types of OST (such as BMT) on BMD, and inclusion of various potential risk factors which may contribute to low BMD in the OST population. Other potential areas include machine learning and artificial intelligence that for bone mineral density outcome prediction, which may include various data such as biomarkers.

## Conclusion

Risk assessment for low BMD should be conducted in the OST population. Guidelines and training of practitioners involving in the OST should be provided to increase the detection of low BMD in the OST population.

## Figures and Tables

**Table 1:** Characteristics of the included studies

First author	Year	Type of study	Country	Number of study population	Type of OST	Gender	Risk assessment score
Gotthardt 10	2016	Cross-sectional	Switzerland	144	MMT (69%), MS (25%), BMT (6%)	Male (100%)	5
Grey 11	2010	Cross-sectional	New Zealand	89	MMT (100%)	Male (58%), Female (42%)	4
Sharma 14	2011	Cohort	US	464	MMT (33%)	Female (100%)	5
Kim 12	2006	Cross-sectional	US	92	MMT (100%)	Male (36%), Female (64%)	4
Milos 13	2011	Case-control	Switzerland	11	MMT (100%)	Female (100%)	6

Abbreviations: BMT: buprenorphine maintenance treatment; MMT: methadone maintenance treatment; MS: morphine sulphate; OST: opioid substitution therapy.

**Table 2:** Factors associated with low bone mineral density in opioid substitution therapy patients

Study	Male	Age	Alcohol	Smoking	Opioid use	Body mass index	Testosterone levels
Gotthardt 10		Nil	Nil^a^	Nil^b^	Nil^c^	✓*	✓*
Grey 11	✓		Nil^a^	Nil^b^			
Sharma 14 †					✓^d^		
Kim 12	✓	Nil	✓*^e^	Nil^f^	Nil^g,h^		
Milos 13					Nil^i^		

**Table 2** represents the association between specific factors identified in a specified study. ✓Means the presence of a significant association between specified factor and low bone mineral density. Nil means that the factor was included in specified study, but no association was found with bone mineral density. An empty cell means the factor was not included in the study. † Methadone use is the only factor included in the table as the study consisted of only one-third of patients who had ever used methadone. *positive association; ^a^ alcohol use; ^b^ current smoking; ^c^ duration of opiate use; ^d^ methadone use; ^e^ years of heavy alcohol use; ^f^ years of smoking; ^g^ heroin recent use and duration; ^h^ methadone dose and duration of treatment; ^i^ duration of heroin and methadone use.
